# The Presence of Real Food Usurps Hypothetical Health Value Judgment in Overweight People^1^,^2^,^3^

**DOI:** 10.1523/ENEURO.0025-16.2016

**Published:** 2016-04-29

**Authors:** Nenad Medic, Hisham Ziauddeen, Suzanna E. Forwood, Kirsty M. Davies, Amy L. Ahern, Susan A. Jebb, Theresa M. Marteau, Paul C. Fletcher

**Affiliations:** 1 Department of Psychiatry, University of Cambridge, Cambridge CB2 0SZ, United Kingdom; 2 Wellcome Trust-MRC Institute of Metabolic Science, University of Cambridge, Cambridge CB2 0QQ, United Kingdom; 3 Cambridgeshire & Peterborough NHS Foundation Trust, Cambridge CB21 5EF, United Kingdom; 4 Department of Psychology, Anglia Ruskin University, Cambridge CB1 1PT, United Kingdom; 5 Department of Psychology, University of Cambridge, Cambridge CB2 3EB, United Kingdom; 6 MRC Human Nutrition Research, Cambridge CB1 9NL, United Kingdom; 7 Nuffield Department of Primary Care Health Sciences, University of Oxford, Oxford OX2 6GG, United Kingdom; 8 Behaviour and Health Research Unit, Institute of Public Health, University of Cambridge, Cambridge CB2 0SR, United Kingdom

**Keywords:** eating behavior, food choices, impulsivity, obesity, subjective value, vmPFC

## Abstract

To develop more ecologically valid models of the neurobiology of obesity, it is critical to determine how the neural processes involved in food-related decision-making translate into real-world eating behaviors. We examined the relationship between goal-directed valuations of food images in the MRI scanner and food consumption at a subsequent *ad libitum* buffet meal. We observed that 23 lean and 40 overweight human participants showed similar patterns of value-based neural responses to health and taste attributes of foods. In both groups, these value-based responses in the ventromedial PFC were predictive of subsequent consumption at the buffet. However, overweight participants consumed a greater proportion of unhealthy foods. This was not predicted by in-scanner choices or neural response. Moreover, in overweight participants alone, impulsivity scores predicted greater consumption of unhealthy foods. Overall, our findings suggest that, while the hypothetical valuation of the health of foods is predictive of eating behavior in both lean and overweight people, it is only the real-world food choices that clearly distinguish them.

## Significance Statement

Do overweight people make unhealthier food choices than lean people because they value the healthiness of foods less than lean people do? We show that fMRI markers of valuation of the healthiness of foods do not differ between the lean and overweight groups. While these markers do predict healthy food choices at an *ad libitum* buffet, they do not account for an overall greater selection of unhealthy food choices in the overweight group. This suggests that a fundamental shift in obesity may lie in how the presence of food overcomes prior value-based decision-making.

## Introduction

It is recognized that a major driver of excess weight gain operates at the higher cognitive levels that control eating behavior rather than at the level of metabolic regulation. It is important therefore to develop a more sophisticated understanding of the neural bases of food valuation and choice. Data from epidemiological and laboratory studies suggest that obesity is associated with a greater consumption of foods with high sugar and/or fat content (Hooper et al., 2012; Malik et al., 2013; Morenga et al., 2013) or high energy density (Johnson et al., 2009), all of which are widely perceived as unhealthy (Roberts and Marvin, 2011). This does not seem to be driven by differences in the perception of the healthiness of foods between lean and overweight people (O’Brien and Davies, 2007). This raises the following key question: is obesity associated with a fundamental change in the processes of valuation, such that the consideration of the healthiness of foods plays a smaller role in their valuation in people who are overweight than in people who are lean?

There is robust evidence for the existence of food-related goal value signals in the brain (Bartra et al., 2013; Clithero and Rangel, 2014), but there are two key limitations of these data. First, there is no evidence that neural responses associated with the subjective valuation of foods presented in the experimental setting of the MRI scanner correlate with real-world eating behavior outside the scanner. This is necessary to demonstrate if we are to use within-scan measures as surrogates of real-world food valuation, and as predictors of eating behavior. Second, it is not known whether this valuation process differs in relation to weight status.

Alternatively, maladaptive eating in people who are overweight might not be driven by reduced valuation of the healthiness of foods. This would be consistent with the findings of large-scale surveys that report a high importance attached to the goal of healthy eating for the vast majority of the population, but persistent discrepancies between food intake and dietary recommendations for health (UK Food Standards Agency, 2009). Maladaptive food choices and susceptibility to the development of obesity have been linked to the personality trait of impulsivity (French et al., 2012), which is characterized by a reduced ability to inhibit prepotent responses, and a greater tendency to act without forethought, potentially leading to behaviors that might be in conflict with our goals and values.

Distinguishing between these possibilities will contribute to a fuller understanding of the neurobiology of obesity and may identify new targets for intervention. In this study, we set out to explore whether the extent to which subjective ratings of the healthiness of foods contribute to the neural computation of the goal value of foods (health valuation) is predictive of food choices in a buffet lunch served after the scanning session. We predicted that overweight participants would choose fewer healthy foods, and more unhealthy foods at the buffet, and we sought to investigate whether neural indices of value predicted choice behavior and distinguished between lean and overweight people.

## Materials and Methods

### Participants

We recruited 69 healthy, right-handed participants (mean age, 30.1 years; SD 6.1 years; age range, 18-40 years; mean BMI, 27.9 kg/m^2^; SD, 5.9 kg/m^2^; BMI range, 19.9-44.5 kg/m^2^; 39 females) in the following two groups: lean (BMI, <25 kg/m^2^) and overweight (BMI, >= 25 kg/m^2^), matched for age, gender, education, income, and IQ. All participants had normal or corrected-to-normal vision, had no history of psychiatric or other significant medical history, and reported no contraindications to MRI scanning. Engaging in a high-intensity workout >3 h/week was also one of the exclusion criteria; the reason for including the limit of weekly exercise was to exclude athletes whose BMI would, due to increased muscle mass, falsely classify them as overweight. Furthermore, we excluded vegetarians and people with any other specific dietary preferences or allergies relating to the food items used in the study. Particular effort was invested to make the sample of participants representative of the U.K. population and participants were recruited from the wider community rather than exclusively from the University of Cambridge. Specifically, given that a greater prevalence of overweight and obesity is found in lower socioeconomic groups (Department of Health Public Health Research Consortium et al., 2007; National Obesity Observatory, 2012), effort was made to recruit groups of lean and overweight people with an overall comparable variability of education levels and yearly incomes (in order to dissociate the adiposity-linked differences in food choices and valuation from the potential confound of socioeconomic status).

The study was approved by the [Author University] Psychology Research Ethics Committee and was conducted at two departments of University of Cambridge. The study was performed in accordance with the principles of the Declaration of Helsinki. All participants provided written, informed consent.

Six participants were excluded from the analysis, as follows: three participants did not complete the study, and the behavioral data were inadvertently not saved for two participants, which prevented the analysis of their fMRI data. One participant was involved in rigorous physical training (bodybuilding), which was not detected during the screening process. The demographics of the remaining 63 participants (23 lean and 40 overweight participants), whose data were processed and analyzed, are presented in Table 1.

**Table 1.** Study sample demographics

Measure	Lean (*n* = 23)	Overweight/obese (*n* = 40)	*t*/χ^2^	*p*
BMI (kg/m^2^)	21.88 (1.3)	30.84 (4.82)	8.70	<0.001
Age (years)	29.78 (6.00)	29.85 (5.75)	0.04	0.97
Gender				
Female	13	23	0.01	0.99
Male	10	17		
Education				
University degree	13	21	0.01	0.96
No university degree	10	19		
Average yearly income (£)				
≤9,999	7	11	2.41	0.49
10,000–19,999	10	13		
20,000–29,999	3	12		
30,000–39,999	3	3		
Ethnicity				
White	20	35	0.90	0.82
Black	1	2		
Asian	2	2		
Other	0	1		
IQ	107.45 (12.78)	111.28 (17.45)	0.90	0.37
DEBQ				
Restraint	22.86 (8.35)	26.58 (5.87)	2.05	0.05
Emotional	27.23 (8.15)	31.58 (9.58)	1.80	0.08
External	30.73 (4.58)	32.45 (6.15)	1.15	0.26

Values are reported as the mean (SD) or *n*, unless otherwise indicated. DEBQ, Dutch Eating Behavior Questionnaire.

### Study design

**Figure 1.**
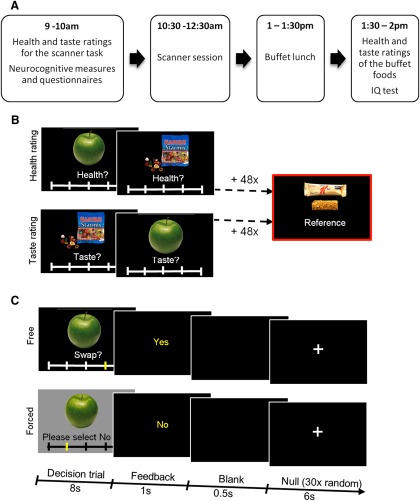
Study design and experimental task. ***A***, Study design. ***B***, Before the scanner session, participants rated 50 foods for their healthiness and tastiness, in two separate ratings blocks, the order of which was counterbalanced across participants. For each participant, the health- and taste-neutral food was selected as the reference food for the scanner task. ***C***, The scanner food choice task featured the same 50 items presented as part of free and forced trials. Free and forced trials, of 8 s duration, were presented in a randomized order. After the decision trial was over, a 1 s feedback screen presented the decision that was made. This was followed by a 0.5 s blank screen. On 30 random occasions during the course of the task, a 6 s null trial with a fixation cross was presented after the blank screen.

Before coming to take part in the study, participants were instructed to eat their standard breakfast at home before 8:00 A.M. All aspects of the study were conducted on a single day in the same order (Fig. 1A). The study session started at 9:00 A.M., after which the health and taste ratings of the scanner task foods were collected, the scanner tasks were thoroughly explained and practiced, and additional cognitive measures were collected. These included tasks that examined response inhibition (Stop Signal Reaction Time (SSRT); Logan, 1994), Stroop interference (SI; Golden and Freshwater, 2002), a self-report questionnaire assessing impulsivity (BIS-11; Patton et al., 1995), and an eating behavior questionnaire (Dutch Eating Behaviour Questionnaire; van Strien et al., 1986). The scanning session started at 10:30 A.M., and the buffet lunch was served from 1:00 to 1:30 P.M. After lunch, subjects rated the healthiness and taste of the foods offered to them in the buffet, and completed an IQ test (test of G; Cattell and Cattell, 1950).

### The food choice task

The task used to explore food valuation was based on Hare et al. (2009). Prior to the scanning session, participants rated 50 food items (common snack foods), presented on a computer screen, on a 5-point scale for their healthiness (Very Unhealthy, Unhealthy, Neutral, Healthy, and Very Healthy, coded in the behavioral and fMRI analysis as 1, 2, 3, 4, and 5, respectively) and tastiness (Very Bad, Bad, Neutral, Good, and Very Good, coded in the behavioral and fMRI analysis as 1, 2, 3, 4, and 5, respectively). This was conducted in two separate blocks, the order of which was counterbalanced across participants (Fig. 1B). Before the taste-rating block, participants were instructed to “rate the tastiness of each food item without regard for its healthiness,” and, correspondingly, before the health-rating block they were instructed to “rate the healthiness of each food item without regard for its tastiness.”

Following the two rating blocks, one item that was rated as neutral on both health and taste scales was selected as the reference food item for that participant (for participants who did not have an item rated as neutral on both scales, we selected an item that was rated neutral on the taste scale and healthy on the health scale as the reference item). Given that the reference item was kept consistent throughout for each participant, the valuation was ultimately expressed with reference to this individually specific constant.

Participants were shown a picture of the reference food item at the beginning of the task and told that on each trial they would have to choose between the food item shown on that trial and the reference food item (Fig. 1C). They were told to imagine that each offered swap constitutes a real food choice, and to treat each swap as if it were the only one offered. We note that, in contrast with the task used by Hare et al. (2009), and due to our overall study design, which included a buffet lunch, our in-scanner food choices were completely hypothetical. To indicate how willing they would be to accept the swap, participants selected (on a sliding scale below the picture of the offered food) among the following five options: Strong No, No, Neutral, Yes, and Strong Yes, which was taken as a behavioral measure of goal value, and coded in the behavioral and fMRI analysis as 1, 2, 3, 4, and 5, respectively.

Since each trial presented a food stimulus (offered to be swapped for the reference food) and therefore entailed a number of perceptuomotor components, we included control trials [in keeping with previous work (Plassmann et al., 2007; Medic et al., 2014)]. In the control task, the same 50 foods were presented in “forced” trials (as opposed to the “free” trials), in which participants were instructed to select one of five responses that were randomly shown on the screen (“Please select ‘Strong No’/‘No’/‘Neutral’/‘Yes’/‘Strong Yes’”). These trials required participants to engage in all of the processes involved in the free trials, with the critical difference of requiring no subjective valuation. Thus, the aim was to match the free and forced trials as closely as possible, with the exception that the former required participants to indicate the relative value of the food by indicating how willing they were to swap it for the reference item.

Altogether, 50 trials of each trial type (free and forced), with a duration of 8 s, were presented in a randomized order. The picture of the food was presented throughout the entire 8 s duration of the trial. The initial position of the cursor on the sliding scale varied randomly among all of the five positions of the scale. Participants made responses using a standard button box, with the first and second buttons serving to move the cursor down or up the sliding scale, and the third button serving to confirm their response. Once the confirmation button had been pressed, the cursor could not be moved further until the next trial. When the 8 s trial was over, a feedback screen showing the final decision was presented. If the response was not confirmed within 8 s, the feedback screen stated “Not quick enough.” In the analysis, these trials were considered to be missed trials.

### Buffet

Following the scanning session, participants were provided with an *ad libitum* buffet lunch consisting of a range of sweet and savory foods that were previously rated as healthy and unhealthy by an independent panel and pair matched for energy densities (Table 2). After participants had finished eating, the remaining food was weighed.

**Table 2.** Foods comprising the buffet lunch

Food	kcal/100 g	Fat/100 g	Saturated fat/100 g	Weight/volume as served (g)	Calories available
Cheddar crackers	509	27.7	16.0	200	1018
Oatcake crackers	449	21.8	8.4	200	898
Chocolate mini bites	440	19.8	3.5	200	880
Eat natural cereal bar	456	24.7	16.4	200	912
Fruit pastille sweets	330	Trace		100	330
Dried mixed fruit	280	0.6	0.2	100	280
Scotch eggs	235	15.3	8.0	400	940
Broccoli and tomato quiche	215	13.2	4.3	400	860
BLT sandwich	225	10.0	2.2	354	797
Chicken salad sandwich	195	7.5	1.0	400	780
Trifle	160	5.4	3.4	600	960
Strawberry yogurt	111	2.6	1.7	600	666
Coke	42			1 L	420
Orange juice	48			1 L	480
Diet coke				1 L	
Water				1 L	

### fMRI analysis

fMRI data were analyzed in spm8, using three models to examine distinct experimental questions. First, we sought to identify the brain circuitry involved in the valuation of the presented food; second, we explored the relationship between prescan health and taste ratings and the neural responses related to valuation. Additionally, in the third model, we investigated group differences in the BOLD signal during food valuation.

In model 1, separate regressors were created for free and forced trials. Free and forced behavioral measures of value (i.e., willingness to accept the swap) were used as parametric modulators of these regressors. To examine the processes specifically associated with valuation, we calculated the first-level contrasts as the difference between the free and forced parametric modulators. To determine which brain regions are involved in valuation across all participants, at the second-level analysis, we computed a one-sample *t* test on the single-participant contrast coefficients from all participants.

In model 2, we investigated the extent to which the health and taste ratings contributed to neural activity underlying goal value computation. We therefore restricted our analysis to the value-coding cluster established in the previous analysis (goal value coding functional ROI). Health and taste ratings of the foods were used as parametric modulators of the free trial regressors. To determine the contribution of each individual’s health and taste ratings to their pattern of neural activity associated with goal value computation, we extracted individual-level health and taste beta values from the individual peak goal value-coding voxels within the value-coding functional ROI. To validate the results of this fMRI analysis, we additionally estimated the degree to which each participant’s health and taste ratings contributed to the behavioral measure of the value inferred from food swaps.

In model 3, we explored the group differences in the BOLD response during food valuation. To examine the BOLD response specifically related to valuation, we calculated the first-level contrast as the difference in BOLD responses between the free and forced trials. To examine the differences between lean and overweight participants, we conducted two *t* tests (lean < overweight, overweight > lean) on the first-level contrast estimates. We restricted our analysis to the previously defined goal value-coding functional ROI, and also explored the existence of significant clusters across the whole brain.

### Statistical analyses and model visualization

Behavioral data were analyzed using linear models (lm package in R) and linear mixed-effects models (nlme package; Pinheiro et al., 2013), in which participants were modeled as a random effect. To perform stepwise linear model selection, we used the stepAIC function, available in the MASS package (Venables and Ripley, 2002). Fitted linear multiple regression models (Fig. 4) were visualized using the visreg function (package visreg). Cross-validation of the multiple regression models was performed using the CVlm function (package DAAG). Further details of the statistical analyses can be found in Table 7 (superscript letters in the Results section, figure and table legends refer to statistical results listed in Table 7).

## Results

### Behavioral results

#### Food choice task

**Figure 2.**
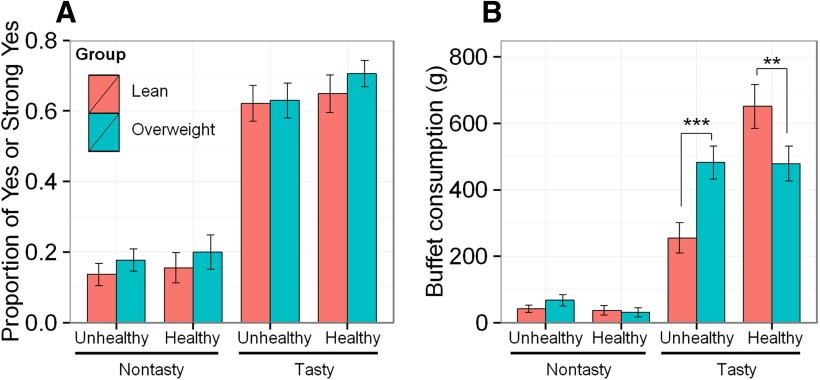
Food choices in the scanner task and in the buffet lunch. ***A***, The proportion of acceptance of food swaps (selecting “yes” or “strong yes”) in the scanner food choice task, across four categories of foods, in lean participants (*n* = 23) and overweight participants (*n* = 40). ***B***, Buffet consumption (expressed as the weight of consumed foods) across four food categories, in lean and overweight participants. **p < 0.01, ***p < 0.001. Error bars represent the SEM.

Lean and overweight participants did not differ in their health ratings for the food items (*t*_(61)_ = −1.47, *p* = 0.15^a^), suggesting a similar perception of healthiness of these foods. They also did not differ in their taste ratings for the same food items (*t*_(61)_ = 1.22, *p* = 0.23^b^). Based on individual health and taste ratings, foods were classified as healthy or unhealthy (health factor), and as tasty or nontasty (taste factor), resulting in four food categories (healthy-tasty, healthy-nontasty, unhealthy-tasty, and unhealthy-nontasty); given that the categorization of foods was performed separately for each participant, based on their individual ratings, foods representing each category differed across participants. Per each participant, foods were designated as tasty if the tastiness of the food was rated as Very Good or Good; or nontasty if the participant rated the tastiness of food as Neutral, Bad, or Very Bad. Analogously, based on the health ratings, each food was designated as either healthy, if the healthiness of the food was rated as Very Healthy or Healthy, or unhealthy, if the participant rated the healthiness of that food as Neutral, Unhealthy, or Very Unhealthy. We estimated a linear mixed-effects model to explore the effect of the health and taste factors, and group (lean and overweight), on the proportion of swaps accepted (Yes or Strong Yes). The analysis revealed a single main effect of the taste factor (*F*_(1,180)_ = 309.11, *p* < 0.0001^c^), with participants accepting more swaps for tasty than nontasty foods (Fig. 2A). An analogous analysis of the time taken to decide about the swap as a function of the health and taste factors, and group, found no significant main or interaction effects^d^.

#### Neurocognitive measures of impulsivity

We examined the differences between lean and overweight participants for three measures of impulsivity, namely, SSRT^e^, SI^f^, and the self-report questionnaire BIS-11^g^. None of these measures differed between lean and overweight participants (Table 3).

**Table 3.** Mean scores of neurocognitive measures of impulsivity in lean and overweight participants

Measure	Lean	Overweight	*t*	*p*
SSRT (*n* = 61)	161.09 ms (39.5 ms)	172.1 ms (58 ms)	−0.80	0.43^e^
SI (*n* = 62)	229.03 ms (231.07 ms )	243.71 ms (249.23 ms)	0.23	0.82^f^
BIS-11 (*n* = 63)	66.74 (7.79)	62.3 (9.11)	1.96	0.06^g^

Values are reported as the mean (SD), unless otherwise indicated.

#### Buffet consumption

To increase specificity, per each participant, buffet foods were categorized based on individual health and taste ratings, and, following the same protocol as that used with the scanner foods, into healthy and unhealthy, and tasty and nontasty (the participants’ health and taste ratings were overall closely aligned with the ratings of the panel). For each participant, we summed consumption (in grams) for each of the four food categories. We then estimated a linear mixed-effects model to explore the effect of the health and taste factors, and group (lean and overweight), on the weight of the food consumed. This analysis revealed a main effect of taste factor (*F*_(1,169)_ = 219.13, *p* < 0.0001^h^) and a smaller, but significant, effect of health factor (*F*_(1,169)_ = 4.35, *p* = 0.04^h^) on consumption (Fig. 2B). The group factor did not affect consumption (*F*_(1,60)_ = 0.29, *p* = 0.59^h^), demonstrating that, overall, lean and overweight participants did not differ in their total consumption. However, they differed in their food choices within the buffet, as follows: consumption was significantly influenced by a three-way interaction among the health and taste of foods and group (*F*_(1,169)_ = 9.29, *p* = 0.003^h^). This interaction was driven by significant health-by-taste interactions (*F*_(1,169)_ = 8.23, *p* = 0.005^h^) and health-by-group interactions (*F*_(1,169)_ = 13.09, *p* < 0.001^h^). Tukey’s *post hoc* tests within the four food categories revealed that lean participants consumed significantly more healthy-tasty foods than the overweight participants (*p* = 0.005), while the overweight participants consumed significantly more unhealthy-tasty foods than the lean participants (*p* < 0.001). Similar results were seen when consumption was examined separately for solid foods^i^ and drinks^j^.

Additionally, we conducted a linear mixed-effects analysis of the energy intake at the buffet, in a manner analogous to the analysis of the weight of consumed foods. Similarly, as with the analysis of consumed weight, this analysis revealed main effects of the taste of foods (*F*_(1,169)_ = 137.84, *p* < 0.0001^k^) and the health of foods (*F*_(1,169)_ = 16.2, *p* = 0.0001^k^) on energy intake. The group factor on its own did not affect energy intake (*F*_(1,60)_ = 0.26, *p* = 0.61^k^). However, energy intake was significantly influenced by a three-way interaction among the health and taste factors and group (*F*_(1,169)_ = 9.98, *p* = 0.002^k^). This interaction was driven by significant health-by-taste interactions (*F*_(1,169)_ = 4.76, *p* = 0.03^k^) and health-by-group interactions (*F*_(1,169)_ = 11.86, *p* < 0.001^k^). Tukey’s *post hoc* tests within the four food categories revealed that the lean participants consumed significantly more energy from healthy-tasty foods than the overweight participants (*p* = 0.004), while the overweight participants consumed significantly more calories from unhealthy-tasty foods than the lean participants (*p* < 0.001).

### fMRI results

As described above, three analyses were performed. The first analysis sought to identify regions involved in the computation of goal value. In the second analysis, we examined the extent to which taste and health attributes contributed to the neural computation of goal value. In the third analysis, we explored group differences in the BOLD signal during food valuation.

#### Model 1: brain circuitry involved in goal valuation

**Figure 3.**
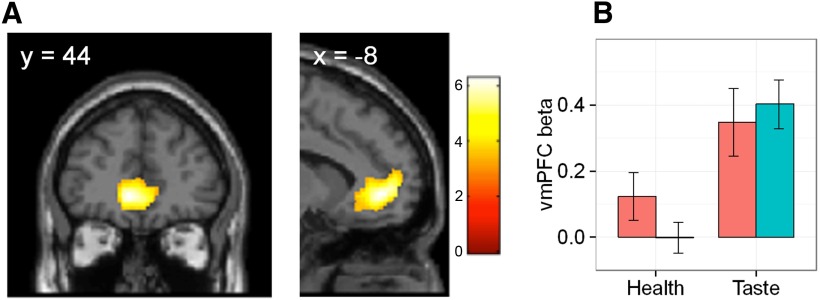
Neural measures of the goal value of food. ***A***, The neural representation of goal value in the vmPFC. The results of the fMRI analysis were rendered onto a standard SPM8 T1 template image, with coronal and sagittal sections presented at the coordinates appropriate for displaying the vmPFC cluster (*p*_FWE_ < 0.05, corrected at the cluster level; *p* < 0.001 uncorrected threshold). ***B***, Health and taste beta value extracted from the vmPFC activity, in lean and overweight participants. Error bars represent the SEM.

As expected from previous work (Bartra et al., 2013; Clithero and Rangel, 2014), the strongest goal value signal was detected in the activity of the ventromedial prefrontal cortex (vmPFC; *p* < 0.05, FWE corrected for multiple comparisons at the cluster level; Fig. 3A). Further, activity correlating with goal value was found in the regions of the posterior cingulate cortex and cuneus (Table 4). For completeness, we conducted two additional analyses. First, we explored the correlation of neural activity with free and forced decisions separately. Whereas the neural activity correlating with free decision strength in free trials mimicked the pattern of neural activity in the main contrast, there was no region, even at a liberal threshold of *p* < 0.001 uncorrected, whose activity correlated with forced decision strength in forced trials. This confirms that the effects established in the main contrast were not driven by activity associated with forced trials. Second, we investigated whether there was a region whose activity tracked the mismatch between free decision and the randomly ascribed forced decision for the same food item during forced trials. In other words, we examined whether being forced to make decisions that deviated from how one would normally make decisions in relation to a given food item was associated with enhanced responses. However, no such region was detected, even at a liberal threshold of *p* < 0.001 uncorrected.

**Table 4.** Brain regions correlated with goal value

Region	Side	Cluster size (voxels)	Peak MNI coordinates			Peak scores	
			*x*	*y*	*z*	*T*	*Z*
Medial frontal gyrus	L/R	1556	−8	44	−4	6.3	5.55
Cuneus	R	663	18	−92	20	5.25	4.78
Posterior cingulate	L/R	544	−8	−46	36	4.48	4.16

*p* < 0.05 whole-brain FWE correction for multiple comparisons at the cluster level (*p* < 0.001 uncorrected threshold).

#### Model 2: the contribution of health and taste attributes to goal value computation

In the second analysis, the value-coding cluster in the vmPFC established in the previous analysis was used as a functional ROI, given its most consistent association with goal value computation in the literature. To determine the contribution of health and taste attributes to the neural activity associated with goal value computation, we extracted individual-level health and taste beta values from the individual peak goal value-coding voxels within the vmPFC functional ROI.

Individual-level taste beta values for this sample were significantly greater than zero (*t*_(62)_ = 6.42, *p* < 0.0001^l^; Fig. 3B), indicating a significant contribution of taste rating to the neural activity in the vmPFC. In contrast, the health ratings of foods on average did not predict neural activity in the vmPFC (*t*_(62)_ = 0.88, *p* = 0.38^m^; Fig. 3B), though there was considerable interindividual variability [coefficient of variation (CV) = 800, compared with a CV of 123.68 for the taste β value]. Furthermore, in a linear mixed-effects model exploring the effect of attribute (health and taste) and group (lean and overweight) on the magnitude of neural beta value, a significant main effect of attribute was established (*F*_(1,61)_ = 23.24, *p* < 0.0001^n^), with neural taste beta value being significantly greater than the neural health beta value in both lean and overweight participants. No main effect of group (*F*_(1,61)_ = 0.21, *p* = 0.65^n^) or attribute-by-group interaction (*F*_(1,61)_ = 1.54, *p* = 0.22^n^) was detected. A separate, single-attribute analysis revealed that neither health (*t*_(61)_ = −1.69, *p* = 0.09^°^) nor taste β value (*t*_(61)_ = 0.45, *p* = 0.66^p^) differed between the groups. Additionally, given the significant interaction between the BIS-11 measures of impulsivity and food consumption in the buffet (see below), we expanded the current model of neural beta values by including BIS-11 scores. While the attribute remained a significant predictor of neural beta values (*F*_(1,59)_ = 22.5, *p* < 0.0001^q^), no other main or interaction effects were detected^q^.

To validate the analysis of the neural beta value, the contributions of health and taste attributes of foods to the behavioral measure of the goal value of food (i.e., the behavioral health and taste beta values) were extracted separately for each participant. Across all of the participants, the mean taste beta value was significantly greater than zero (*t*_(62)_ = 21.53, *p* < 0.0001^r^), whereas the mean health beta value was not significantly different from zero (*t*_(62)_ = 1.92, *p* = 0.06^s^). The behavioral analysis therefore replicated the results of the fMRI analysis in showing that the taste attribute, but not the health attribute, was a significant contributor to the goal valuation of foods. Furthermore, in a linear mixed-effects model exploring the effect of attribute (health, taste) and group (lean, overweight) on the magnitude of the behavioral beta value, results analogous to those from the analysis of the neural beta value were obtained, as follows: a significant main effect of attribute was established (*F*_(1,61)_ = 100.92, *p* < 0.0001^t^), with behavioral taste beta value significantly greater than the health beta value in both lean and overweight participants. No main effect of group (*F*_(1,61)_ = 0.52, *p* = 0.47^t^) or attribute-by-group interaction (*F*_(1,61)_ = 0.01, *p* = 0.94^t^) was detected. A separate, single-attribute analysis revealed that the beta value of neither health (*t*_(61)_ = −0.39, *p* = 0.69^u^) nor taste (*t*_(61)_ = −0.73, *p* = 0.47^v^) differed between the groups. Similar to the case of the neural beta value, the inclusion of BIS-11 as an additional predictor did not explain more variance in the behavioral beta value. The attribute remained a significant predictor of behavioral beta value (*F*_(1,59)_ = 100.9, *p* < 0.0001^w^), while no other main or interaction effects were detected^w^.

#### Model 3: exploring group differences in BOLD response during valuation

Additionally, we investigated the group differences in the BOLD response during valuation. We conducted an ROI-based analysis in the vmPFC functional ROI, and explored the existence of significant clusters at the whole-brain level. *t* Tests, exploring the difference between lean and overweight participants (lean > overweight, overweight > lean) failed to a find significant activation in the vmPFC (*p* = 0.025, FWE small volume correction, Bonferroni corrected for two tests), or any significant clusters at the whole-brain level (*p* < 0.025, FWE corrected for multiple comparisons at the cluster level, Bonferroni corrected for two tests).

### Model of healthy food consumption

Finally, we explored whether the pattern of food consumption in the buffet could be predicted by the individual-level neural beta value, and whether this relationship was modulated by group. Further, we examined whether the inclusion of measures of impulsivity in such a model would capture more variance in the buffet food consumption.

Given that the greatest variability in food consumption across all participants was driven by the health attribute of foods, we used the proportion of healthy foods consumed in the buffet as our main outcome variable (i.e., the consumed weight of foods individually perceived as healthy as a proportion of the total consumption of all foods). We conducted a linear multiple-regression analysis in two stages, performing a stepwise model selection at each stage. We used the stepAIC function implemented in the MASS package in R, which selects the best model fit by minimizing the Akaike information criterion (AIC; Venables and Ripley, 2002). Both a forward and a backward model selection were used, allowing for interactions among variables. To reduce collinearity, all of the continuous predictors were mean centred.

In the first stage of this analysis, the neural health beta and group (overweight − lean) were included as predictors of the proportion of healthy foods consumed. The stepwise procedure returned a model in which the neural health beta value and group were identified as independent, noninteracting predictors of the proportion of healthy foods consumed (Table 5, model 1). The model captured 22.09% of the variance of healthy food consumption (*F*_(2,59)_ = 9.65, *p* < 0.001); the 10-fold cross-validation of the model returned a mean square of prediction error (ms) of 0.0596. The neural health beta value positively predicted the proportion of healthy foods consumed across all participants (β = 0.26, *p* = 0.03^x^); however, over and above this association, the overweight participants consumed a significantly smaller proportion of healthy foods (i.e., a greater proportion of unhealthy foods) than the lean participants (β = −0.37, *p* = 0.002^x^).

**Table 5.** Regression coefficients and corresponding *p* values of the best-fitting models of healthy food consumption in the buffet, as a function of neural health β value, group, and impulsivity scores

Predictor	β	*p*
Model 1^x^		
Neural health beta value	0.26	0.03
Group (overweight − lean)	−0.37	0.002
Model 2^y^		
BIS-11	0.04	0.83
Neural health beta value	0.22	0.03
Group (overweight − lean)	−0.47	<0.001
BIS-11:Group (overweight − lean)	−0.43	0.02

^x^ *F*_(2,59)_ = 9.65, *p* < 0.001; *R* ^2^ = 0.22, ms = 0.0596.

^y^ *F*_(4,55)_ = 12.12, *p* < 0.000; *R* ^2^ = 0.43, ms = 0.0451.

**Figure 4.**
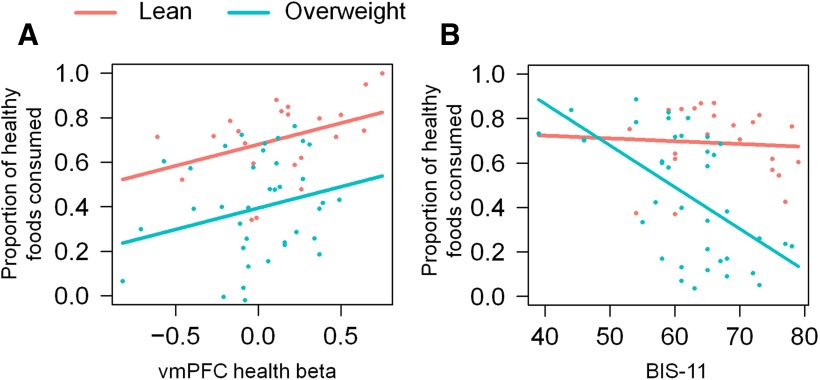
Model of healthy food consumption. Visual depiction of the multiple linear regression model 2 (Table 2). ***A***, A partial residual plot of the proportion of healthy foods consumed as a function of the neural health beta value, in lean and overweight participants. ***B***, A partial residual plot of the proportion of healthy foods consumed as a function of BIS-11 impulsivity scores, in lean and overweight participants. Each dot represents one participant.

In the second stage of the analysis, in addition to the predictors above, we included the following three measures of impulsivity: SSRT, SI, and BIS-11 scores. In this case, the stepwise procedure revealed a best-fitting model that explained 43% of the variance of healthy food consumption (*F*_(4,55)_ = 12.12, *p* < 0.0001; Table 5, model 2; Fig. 4A,*B*), with the cross-validation ms = 0.0451. The neural health beta value (β = 0.22, *p* = 0.03^y^) and group (β = −0.47, *p* < 0.0001^y^) remained as significant independent predictors of the proportion of healthy food consumed (Fig. 4A). Only the BIS-11 remained as a measure of impulsivity in the best-fitting model, and there was a significant interaction between BIS-11 impulsivity scores and group (β = −0.43, *p* = 0.02^y^). In overweight participants, increasing BIS-11 impulsivity was predictive of a smaller proportion of healthy foods consumed (i.e., greater consumption of unhealthy foods), but there was no such association in the lean participants (Fig. 4B).

To validate the above models, the same model procedures were repeated substituting the neural health beta value with the behavioral health beta value, and these resulted in analogous best-fitting models, with similar parameter estimates^z,α^ (Table 6). The analogous analysis for the proportion of tasty food consumption, with neural or behavioral taste beta value, and all other predictors as above, failed to find a significant model of tasty food consumption predicted by any combination of these variables.

**Table 6.** Regression coefficients and corresponding *p* values of the best fitting models of healthy food consumption in the buffet, as a function of behavioral health β value, group and impulsivity scores

Predictor	β	*P*
Model 1^z^		
Behavioral health beta value	0.44	<0.0001
Group (overweight − lean)	−0.4	<0.001
Model 2^α^		
BIS-11	0.04	0.81
Behavioral health beta value	0.26	0.03
Group (overweight − lean)	−0.47	<0.001
BIS-11 group (overweight − lean)	−0.41	0.02

^z^ *F*_(2,59)_ = 17.61, *p* < 0.0001, *R* ^2^ = 0.35, ms = 0.0521.

^α^ *F*_(4,55)_ = 12.3, *p* < 0.0001, *R* ^2^ = 0.43, ms = 0.0457.

**Table 7.** Statistical table

Test	Data structure	Type of test	Test statistic	*p* value	[Confidence interval]/power
a: Overweight − lean	Normal distribution	Linear mixed-effects model	*t*_(61)_ = −1.47	0.15	[−0.25, 0.04]
b: Overweight – lean	Normal distribution	Linear mixed-effects model	*t*_(61)_ = 1.22	0.23	[−0.09, 0.37]
c: Main effect of taste	Normal distribution	Linear mixed-effects model	*F*_(1,180)_ = 309.11	< 0.0001	1
c: Main effect of health	Normal distribution	Linear mixed-effects model	*F*_(1,180)_ = 2.78	0.1	0.39
c: Main effect of group	Normal distribution	Linear mixed-effects model	*F*_(1,61)_ = 0.74	0.39	0.14
c: Health × taste interaction	Normal distribution	Linear mixed-effects model	*F*_(1,180)_ = 0.51	0.48	0.11
c: Health × group interaction	Normal distribution	Linear mixed-effects model	*F*_(1,180)_ = 0.2	0.66	0.07
c: Taste × group interaction	Normal distribution	Linear mixed-effects model	*F*_(1,180)_ = 0.03	0.87	0.05
c: Health × taste × group interaction	Normal distribution	Linear mixed-effects model	*F*_(1,180)_ = 0.17	0.68	0.07
d: Main effect of taste	Normal distribution	Linear mixed-effects model	*F*_(1,180)_ = 1.88	0.17	0.28
d: Main effect of health	Normal distribution	Linear mixed-effects model	*F*_(1,180)_ = 0.96	0.33	0.17
d: Main effect of group	Normal distribution	Linear mixed-effects model	*F*_(1,61)_ = 1.74	0.19	0.27
d: Health × taste interaction	Normal distribution	Linear mixed-effects model	*F*_(1,180)_ = 0.37	0.54	0.09
d: Health × group interaction	Normal distribution	Linear mixed-effects model	*F*_(1,180)_ = 0.61	0.43	0.12
d: Taste × group interaction	Normal distribution	Linear mixed-effects model	*F*_(1,180)_ = 2.19	0.14	0.32
d: Health × taste × group interaction	Normal distribution	Linear mixed-effects model	*F*_(1,180)_ = 0.04	0.85	0.05
e: Overweight − lean	Normal distribution	Two-sample *t* test	*t*_(1,59)_ = −0.8	0.43	[−38.4, 16.4]
f: Overweight – lean	Normal distribution	Two-sample *t* test	*t*_(1,60)_ = −0.24	0.81	[−156, 122]
g: Overweight – lean	Normal distribution	Two-sample *t* test	*t*_(1,61)_ = 1.96	0.06	[−0.09, 8.97]
h: Main effect of taste	Normal distribution	Linear mixed-effects model	*F*_(1,169)_ = 219.13	<0.0001	1
h: Main effect of health	Normal distribution	Linear mixed-effects model	*F*_(1,169)_ = 4.35	0.04	0.56
h: Main effect of group	Normal distribution	Linear mixed-effects model	*F*_(1,60)_ = 0.29	0.59	0.08
h: Health × taste interaction	Normal distribution	Linear mixed-effects model	*F*_(1,169)_ = 8.23	0.005	0.83
h: Health × group interaction	Normal distribution	Linear mixed-effects model	*F*_(1,169)_ = 13.09	0.0004	0.96
h: Taste × group interaction	Normal distribution	Linear mixed-effects model	*F*_(1,169)_ = 0.13	0.72	0.07
h: Health × taste × group interaction	Normal distribution	Linear mixed-effects model	*F*_(1,169)_ = 9.29	0.003	0.87
i: Main effect of taste	Normal distribution	Linear mixed-effects model	*F*_(1,162)_ = 135.05	< 0.0001	1
i: Main effect of health	Normal distribution	Linear mixed-effects model	*F*_(1,162)_ = 6.2	0.01	0.71
i: Main effect of group	Normal distribution	Linear mixed-effects model	*F*_(1,60)_ = 0.01	0.97	0.05
i: Health × taste interaction	Normal distribution	Linear mixed-effects model	*F*_(1,162)_ = 0.48	0.49	0.11
i: Health × group interaction	Normal distribution	Linear mixed-effects model	*F*_(1,162)_ = 8.04	0.005	0.82
i: Taste × group interaction	Normal distribution	Linear mixed-effects model	*F*_(1,162)_ = 0.04	0.84	0.05
i: Health × taste × group interaction	Normal distribution	Linear mixed-effects model	*F*_(1,162)_ = 7.06	0.009	0.77
j: Main effect of taste	Normal distribution	Linear mixed-effects model	*F*_(1,92)_ = 59.26	< 0.0001	1
j: Main effect of health	Normal distribution	Linear mixed-effects model	*F*_(1,92)_ = 41.04	< 0.0001	1
j: Main effect of group	Normal distribution	Linear mixed-effects model	*F*_(1,60)_ = 1.1	0.29	0.19
j: Health × taste interaction	Normal distribution	Linear mixed-effects model	*F*_(1,92)_ = 1.52	0.22	0.24
j: Health × group interaction	Normal distribution	Linear mixed-effects model	*F*_(1,92)_ = 3.21	0.08	0.44
j: Taste × group interaction	Normal distribution	Linear mixed-effects model	*F*_(1,92)_ = 0.59	0.44	0.12
j: Health × taste × group interaction	Normal distribution	Linear mixed-effects model	*F*_(1,92)_ = 2.52	0.12	0.36
k: Main effect of taste	Normal distribution	Linear mixed-effects model	*F*_(1,169)_ = 137.84	<0.0001	1
k: Main effect of health	Normal distribution	Linear mixed-effects model	*F*_(1,169)_ = 16.2	0.0001	0.98
k: Main effect of group	Normal distribution	Linear mixed-effects model	*F*_(1,60)_ = 0.26	0.61	0.08
k: Health × taste interaction	Normal distribution	Linear mixed-effects model	*F*_(1,169)_ = 4.76	0.03	0.59
k: Health × group interaction	Normal distribution	Linear mixed-effects model	*F*_(1,169)_ = 11.86	0.0007	0.94
k: Taste × group interaction	Normal distribution	Linear mixed-effects model	*F*_(1,169)_ = 0.05	0.83	0.06
k: Health × taste × group interaction	Normal distribution	Linear mixed-effects model	*F*_(1,169)_ = 9.98	0.002	0.89
L	Normal distribution	One-sample *t* test	*t*_(62)_ = 6.42	<0.0001	[0.26, 0.5]
M	Normal distribution	One-sample *t* test	*t*_(62)_ = 0.88	0.38	[−0.04, 0.12]
n: Main effect of attribute	Normal distribution	Linear mixed-effects model	*F*_(1,61)_ = 23.24	<0.0001	0.99
n: Main effect of group	Normal distribution	Linear mixed-effects model	*F*_(1,61)_ = 0.21	0.65	0.07
n: Attribute × group interaction	Normal distribution	Linear mixed-effects model	*F*_(1,61)_ = 1.54	0.22	0.24
o: Overweight − lean	Normal distribution	Two-sample *t* test	*t*_(61)_ = −1.69	0.09	[−0.03, 0.3]
p: Overweight − lean	Normal distribution	Two-sample *t* test	*t*_(61)_ = 0.45	0.66	[−0.3, 0.19]

q: Main effect of attribute	Normal distribution	Linear mixed-effects model	*F*_(1,59)_ = 22.5	<0.0001	0.99
q: Main effect of group	Normal distribution	Linear mixed-effects model	*F*_(1,59)_ = 0.2	0.65	0.07
q: Main effect of BIS-11	Normal distribution	Linear mixed-effects model	*F*_(1,59)_ =0.01	0.83	0.06
q: Attribute × group interaction	Normal distribution	Linear mixed-effects model	*F*_(1,59)_ = 1.5	0.23	0.24
q: Attribute × BIS-11 interaction	Normal distribution	Linear mixed-effects model	*F*_(1,59)_ = 0.1	0.75	0.06
q: Group × BIS-11 interaction	Normal distribution	Linear mixed-effects model	*F*_(1,59)_ = 0.01	0.93	0.05
q: Attribute × group × BIS-11 interaction	Normal distribution	Linear mixed-effects model	*F*_(1,59)_ = 0.01	0.93	0.05
r	Normal distribution	One-sample *t* test	*t*_(62)_ = 21.53	<0.0001	[0.51, 0.61]
s	Normal distribution	One-sample t-test	*t*_(62)_ = 1.92	0.06	[0, 0.15]
t: Main effect of attribute	Normal distribution	Linear mixed-effects model	*F*_(1,61)_ = 100.92	<0.0001	1
t: Main effect of group	Normal distribution	Linear mixed-effects model	*F*_(1,61)_ = 0.47	0.47	0.11
t: Attribute × group interaction	Normal distribution	Linear mixed-effects model	*F*_(1,61)_ = 0.01	0.94	0.05
u: Overweight − lean	Normal distribution	Two-sample *t* test	*t*_(61)_ = −0.39	0.69	[−0.13, 0.19]
v: Overweight − lean	Normal distribution	Two-sample *t* test	*t*_(61)_ = −0.73	0.47	[−0.07, 0.15]
w: Main effect of attribute	Normal distribution	Linear mixed-effects model	*F*_(1,59)_ = 100.9	< 0.0001	1
w: Main effect of group	Normal distribution	Linear mixed-effects model	*F*_(1,59)_ = 0.5	0.47	0.11
w: Main effect of BIS-11	Normal distribution	Linear mixed-effects model	*F*_(1,59)_ = 0.4	0.54	0.1
w: Attribute × group interaction	Normal distribution	Linear mixed-effects model	*F*_(1,59)_ = 0.01	0.94	0.05
w: Attribute × BIS-11 interaction	Normal distribution	Linear mixed-effects model	*F*_(1,59)_ = 3.2	0.08	0.44
w: Group × BIS-11 interaction	Normal distribution	Linear mixed-effects model	*F*_(1,59)_ = 0.2	0.65	0.07
w: Attribute × group × BIS-11 interaction	Normal distribution	Linear mixed-effects model	*F*_(1,59)_ = 0.2	0.67	0.07
x: Neural β value	Normal distribution	Linear model	*t*_(1,59)_ = 2.24	0.03	[0.02, 0.43]
x: Overweight − lean	Normal distribution	Linear model	*t*_(1,59)_ = −3.24	0.002	[−0.35, −0.08]
y: BIS-11	Normal distribution	Linear model	*t*_(1,55)_ = −0.21	0.83	[−0.01, 0.01]
y: Neural β value	Normal distribution	Linear model	*t*_(1,55)_ = 2.21	0.03	[0.02, 0.36]
y: Overweight − lean	Normal distribution	Linear model	*t*_(1,55)_ = −4.35	<0.0001	[−0.39, −0.15]
y: BIS-11 × (overweight − lean) interaction	Normal distribution	Linear model	*t*_(1,55)_ = −2.45	0.02	[−0.03, 0]
z: Behavioral β value	Normal distribution	Linear model	*t*_(1,59)_ = 4.25	< 0.0001	[0.2, 0.57]
z: Overweight − lean	Normal distribution	Linear model	*t*_(1,59)_ = −3.9	0.0003	[−0.36, −0.11]
α: BIS-11	Normal distribution	Linear model	*t*_(1,55)_ = 0.24	0.81	[−0.01, 0.01]
α: Behavioral β value	Normal distribution	Linear model	*t*_(1,55)_ = 2.29	0.03	[0.03, 0.43]
α: Overweight − lean	Normal distribution	Linear model	*t*_(1,55)_ = −4.35	< 0.0001	[−0.39, −0.15]
α: BIS-11 x (overweight − lean) interaction	Normal distribution	Linear model	*t*_(1,55)_ = −2.34	0.02	[−0.03, 0]

## Discussion

Our findings in lean and overweight people offer intriguing insights into food valuation, its relationship to neural signals and impact on decision-making. To summarize, we confirmed that value-based decision-making is related to vmPFC activity, with activity in this region reflecting the goal value of presented foods. The degree to which the health and taste attributes of foods contributed to this vmPFC activity (the neural health and taste beta values) did not differ between lean and overweight participants. Importantly, the contribution of health attributes to the neural value signal was predictive of the proportion of healthy foods consumed in the buffet, demonstrating its validity as a measure of real-world valuation and choice. In both lean and overweight groups, those with a higher health beta value chose a greater proportion of healthy foods, and critically, this relationship did not differ between the groups. This is demonstrated by the similar slopes for the two groups in the graph (Fig. 4A). However, the overall proportion of healthy foods consumed in the buffet was significantly greater in lean participants (i.e., overweight participants consumed a significantly greater proportion of unhealthy foods). This is demonstrated by the differing intercepts for the two groups (Fig. 4A). Our results, therefore, indicate that the increased real-world consumption of unhealthy foods by people who are overweight is not driven by reduced valuation of the healthiness of food, as assessed by subjective or neural responses. Rather, for a given level of such value placed upon health, there is less actual consumption of healthy food in the overweight people. Intriguingly, in the overweight participants, the proportion of healthy foods consumed was further modulated by impulsivity scores: participants who were overweight and highly impulsive consumed the largest proportion of unhealthy foods in the buffet. Below, we consider the implications of these findings.

At the group level, the taste attribute significantly contributed to the neural computation of goal value of foods, which is in line with previous work by Hare et al. (2009), and was also a major factor affecting food choices at the buffet. It is important to note that, while in the scanner food choice task participants made binary forced choices, in the buffet lunch they freely selected foods to consume and unsurprisingly, predominantly chose foods that they rated as tasty. In other words, there was practically no interindividual variability in the proportion of tasty foods consumed in the buffet, which explains why the contribution of the taste attribute to the goal valuation of foods in the vmPFC at the individual level did not predict the individual consumption of tasty foods in the buffet lunch.

In contrast, the health attribute was not, at the group level, a significant contributor to the goal value computation of foods in the vmPFC in either lean or overweight participants. This was because there was, as might be expected, appreciable interindividual variability in the contribution of the health attribute to the goal value computation within each group, with no differences between the groups. Capitalizing on this variability, we show that, in both groups, it predicted the proportion of healthy foods consumed in the buffet. In other words, the neural signal of health valuation—the weight given to the health attribute in the goal value computation of foods—predicts real-world choices. This provides support for the use of such measures in studying goal valuation in relation to eating choices.

However, while the hypothetical choice offered in the scanner produced a neural signal for health valuation that was strongly predictive of subsequent individual-level eating behavior, it did not predict differences between lean and overweight people (Fig. 4A, parallel slopes). Importantly, however, overweight participants ate a significantly smaller proportion of foods they individually regarded as healthy, compared with their lean counterparts (Fig. 4A, difference in intercepts). This suggests that over and above the effect of hypothetical health valuation, which does not differ between the groups, and equally affects their real-world behavior, a real-world bias toward unhealthy foods is present in people who are overweight.

What might drive this effect? One possibility is that a different behavioral construct, other than goal-directed valuation, may mediate the differences between lean and overweight participants in real-world food choices. It is relevant, in this respect, that impulsivity scores showed their effects only in the overweight individuals in the context of actual consumption. In children, impulsivity scores have been linked to greater BMI and greater food consumption; however, this relationship is less clear in adults (French et al., 2012), where several studies suggest that greater impulsivity scores per se do not confer risk to maladaptive eating or obesity. More often, impulsivity scores have been reported to interact with implicit measures of motivation for foods in predicting food intake and obesity (Hofmann et al., 2009; Nederkoorn et al., 2010; Rollins et al., 2010; Epstein et al., 2014). This suggests that the combination of a high motivation for food and a reduced capacity to inhibit prepotent responses act together in raising the risk of overeating and obesity.

According to the theory of incentive salience, such implicit motivation, or “wanting,” can be dissociated from the explicit valuation of rewards and is induced upon encountering rewards, or their associated stimuli, that have previously been experienced as pleasant or liked (Berridge, 2007). Highly palatable foods, which are often perceived as unhealthy, are thus likely to induce the strongest implicit motivation. In line with these theoretical perspectives, there is evidence that such motivation is most strongly induced in the physical presence of rewards (Mischel and Moore, 1973; Bushong et al., 2010; Woelbert and Goebel, 2013), consequently affecting our decisions and often promoting divergence from our goals in many decision-making scenarios, including eating. For example, the expression of such motivation might explain the effects of food cues to increase appetite (Ferriday and Brunstrom, 2008). Critically, its dependence on the physical presence of rewards provides a good conceptual fit to our data, where differences in food choices between lean and overweight participants were only observed in the buffet (i.e. once participants were presented with foods to choose for immediate consumption).

Several studies indicate that the effects of the physical presence of foods on consumption, and the motivation for foods, might be more pronounced in overweight than in lean participants. Schachter and Rodin (1974) argued that overweight participants are more sensitive to external cues of food proximity than lean participants. More recently, it was demonstrated that overweight participants express a comparatively greater motivation/desire for food following exposure to food cues (Tetley et al., 2009; Ferriday and Brunstrom, 2011). Studies exploring the effects of food cues on eating behavior in children demonstrated that overweight children, upon smelling food (Jansen et al., 2003) or watching food TV commercials (Halford et al., 2004), increase their consumption to a greater extent than lean participants. Furthermore, it has been reported that overweight participants are willing to work harder to obtain food rewards (Saelens and Epstein, 1996; Temple et al., 2008).

Another possibility that we should consider is that it is differential valuation that drives differing choices across groups. Indeed, it is known that different choices may be made in the hypothetical compared with the real condition. Despite the demonstration that the same neural circuitry encodes both hypothetical and real decisions (Kang et al., 2011), a number of studies have described a hypothetical bias (i.e. the tendency to overstate hypothetical valuations; List and Gallet, 2001; Little and Berrens, 2004; Murphy et al., 2005). In the study by Kang et al. (2011), while the indifference curves for hypothetical and real choices had the same shape (reminiscent of the parallel slopes in Fig. 4A), the indifference point in the hypothetical condition was shifted toward a larger value. The reported existence of such a bias provides one way of interpreting our data: while we have demonstrated the predictive validity of hypothetical valuation, we acknowledge the possibility that overweight participants might have attributed greater weight to the healthiness of food in the hypothetical than in the real-world condition. We note that, compared with the hypothetical scanner condition, in the buffet, participants were not constrained by limited time to make choices, and were also in a hungrier state, all of which could have been factors that contributed to a change in health valuation in the real condition. Such an account is in line with sequential sampling models of decision-making, which describe valuation as a sequential process in which the recollection of new information or a change in conditions can gradually modify the initial value estimate (Otter et al., 2008). Overall then, the betweengroup difference in food choices in the real versus the hypothetical condition, which we observed here, could reflect group differences in health valuation across the two conditions, as well as differences in the implicit motivation for food, and the extent to which trait impulsivity manifests in the presence of food.

We were only able to study a limited range of foods, and it is not possible to study eating behavior in this detail in naturalistic settings. We cannot be certain how the scanner or the buffet meal affected individual behavior, despite our efforts to create a relaxed eating environment for the latter. One thing is clear: while fMRI signals were meaningful and predictive of real-world behaviors, it was only with the presentation of real food choices that the group differences emerged. The study thus provides an important indication that, while fMRI experiments offer precise and predictive measures of key processes related to value, choice, and consumption, they must be complemented by other, more naturalistic measures.

In summary, we show that the individual variability in the weights given to health attributes in goal value computation of foods in the vmPFC predicts food choices in a buffet lunch. More specifically, we demonstrated that people who are overweight make fewer real-world healthy food choices compared with their lean counterparts, in contrast with the hypothetical condition, where their health valuations of foods are indistinguishable from those of lean participants. While impulsivity did not fully account for these differences, it was striking that, in overweight participants only, increased impulsivity scores were associated with a greater proportion of unhealthy foods consumed. Importantly, these results suggest that the bias toward the consumption of unhealthy foods among participants who are overweight is expressed primarily in the presence of readily available foods. They add further weight to existing evidence that interventions to reduce food consumption in those who are overweight are more likely to be effective when targeted at the processes, often automatic and nonconscious, that get activated by the omnipresence of highly palatable unhealthy foods in our everyday environments.

## Synthesis

The decision was a result of the Reviewing Editor Philippe Tobler and the peer reviewers coming together and discussing their recommendations until a consensus was reached. A fact-based synthesis statement explaining their decision and outlining what is needed to prepare a revision is listed below. The following reviewer(s) agreed to reveal their identity: Alain Dagher

Dear Dr. Medic,

Your manuscript has now been seen by the two two reviewers who reviewed it for JNS. They were largely happy with the revision. However, some points need to be addressed before we can accept the paper.

Kind regards,

Phliippe Tobler

This very nice study tests a clear hypothesis: that obesity is associated with normal valuation of healthiness of foods in the abstract, as shown by subjective ratings and fMRI, but that the proximity of food overcomes these healthiness values when a choice has to be made.

Authors had a large number of subjects (n=63 after exclusions) rate food items for taste and healthiness. Then subjects underwent fMRI with the food auction task popularized by Rangel.

The study is very well done and the number of subjects studied is impressive. However I am not sure the authors have proved their main conclusion. To show that hypothetical evaluation (whether neural of behavioral) had less influence on real-world choices in overweight people, one would have to show that the health related value signal (beta) in vmPFC was less predictive of real choices in that group. But this is not the case:

"In both lean and overweight groups, those with higher health betas chose a greater proportion of healthy foods and this relationship did not differ between the groups."

On the other hand:

"people who are overweight make fewer real-world healthy food choices compared to their lean counterparts, in contrast to the hypothetical condition, where they express a comparable health valuation of foods as lean participants."

These two conclusions argue for different interpretations regarding the relationship between hypothetical and real-world choices in overweight versus lean, do they not?

In other words the findings are as follows:

1) Health betas in vmPFC no different in the two groups

2) Relationship between health betas in vmPFC and real-world choices no different in the two groups.

3) Greater proportion of unhealthy foods consumed in the overweight group.

While #1 and #3 support the hypothesis, doesn't #2 contradict it?

Could the greater proportion of unhealthy foods consumed by the overweight group then not reflect the fact that taste was a greater driver of consumption than healthiness (for all subjects, but more so for overweight)?

Moreover, Figure 3 seems to show that the vmPFC value signal (during the hypothetical food choice) was mostly driven by taste, with a smaller influence of health in lean, and no effect of health in overweight. In other words, isn't this in keeping with the opposite interpretation: healthiness does not drive the value signal (in the hypothetical situation) and does not drive choice (in the real world situation) in the overweight (or does not drive it as much as in the lean).

Put another way: in both groups the neural valuation predicts consumption, but health plays little role in either neural valuation or real world choice in the overweight? While in the lean value is a combination of healthiness and taste.

Note that my suggestion is that this issue be addressed either in a Reply or in the Discussion. Despite this question, this remains a strong contribution to the literature on brain mechanisms of food choice.

Lines 94-95 p.4: extraneous text.

## References

[B1] Bartra O, McGuire JT, Kable JW (2013) The valuation system: a coordinate-based meta-analysis of BOLD fMRI experiments examining neural correlates of subjective value. Neuroimage 76:412–427. 10.1016/j.neuroimage.2013.02.063 23507394 PMC3756836

[B2] Berridge KC (2007) The debate over dopamine’s role in reward: the case for incentive salience. Psychopharmacology (Berl) 191:391–431. 10.1007/s00213-006-0578-x 17072591

[B3] Breheny P, Burchett W Visualization of Regression Models Using visreg. https://cran.r-project.org/web/packages/visreg/visreg.pdf

[B4] Bushong B, King LM, Camerer CF, Rangel A (2010) Pavlovian processes in consumer choice: the physical presence of a good increases willingness-to-pay. Am Econ Rev 100:1556–1571. 10.1257/aer.100.4.1556

[B5] Cattell RB, Cattell AKS (1950) Test of “g” culture fair. Champaign, IL: Institute for Personality and Ability Testing.

[B6] Clithero JA, Rangel A (2014) Informatic parcellation of the network involved in the computation of subjective value. Soc Cogn Affect Neurosci 9:1289–1302. 23887811 10.1093/scan/nst106 PMC4158359

[B7] Department of Health Public Health Research Consortium, Law C, Power C, Graham H, Merrick D (2007) Obesity and health inequalities. Obes Rev 8 [Suppl 1]:19–22. 10.1111/j.1467-789X.2007.00312.x [Mismatch] 17316296

[B8] Epstein LH, Jankowiak N, Fletcher KD, Carr KA, Nederkoorn C, Raynor HA, Finkelstein E (2014) Women who are motivated to eat and discount the future are more obese. Obesity 22:1394–1399. 10.1002/oby.20661 24311480 PMC4007365

[B9] Ferriday D, Brunstrom JM (2008) How does food-cue exposure lead to larger meal sizes? Br J Nutr 100:1325–1332. 10.1017/S0007114508978296 18466651

[B10] Ferriday D, Brunstrom JM (2011) “I just can’t help myself”: effects of food-cue exposure in overweight and lean individuals. Int J Obes 35:142–149. 10.1038/ijo.2010.117 20548302

[B11] French SA, Epstein LH, Jeffery RW, Blundell JE, Wardle J (2012) Eating behavior dimensions. Associations with energy intake and body weight. A review. Appetite 59:541–549. 10.1016/j.appet.2012.07.001 22796186 PMC3454469

[B12] Golden CJ, Freshwater SM (2002) Stroop color and word test: a manual for clinical and experimental uses. Chicago: Stoelting.

[B13] Halford JC, Gillespie J, Brown V, Pontin EE, Dovey TM (2004) Effect of television advertisements for foods on food consumption in children. Appetite 42:221–225. 10.1016/j.appet.2003.11.006 15010186

[B14] Hare TA, Camerer CF, Rangel A (2009) Self-control in decision-making involves modulation of the vmPFC valuation system. Science 324:646–648. 10.1126/science.1168450 19407204

[B15] Hofmann W, Friese M, Roefs A (2009) Three ways to resist temptation: the independent contributions of executive attention, inhibitory control, and affect regulation to the impulse control of eating behavior. J Exp Soc Psychol 45:431–435. 10.1016/j.jesp.2008.09.013

[B16] Hooper L, Abdelhamid A, Moore HJ, Douthwaite W, Skeaff CM, Summerbell CD (2012) Effect of reducing total fat intake on body weight: systematic review and meta-analysis of randomised controlled trials and cohort studies. BMJ 345:e7666. 23220130 10.1136/bmj.e7666 PMC3516671

[B17] Jansen A, Theunissen N, Slechten K, Nederkoorn C, Boon B, Mulkens S, Roefs A (2003) Overweight children overeat after exposure to food cues. Eat Behav 4:197–209. 10.1016/S1471-0153(03)00011-4 15000982

[B18] Johnson L, Wilks DC, Lindroos AK, Jebb SA (2009) Reflections from a systematic review of dietary energy density and weight gain: is the inclusion of drinks valid? Obes Rev 10:681–692. 10.1111/j.1467-789X.2009.00580.x 19413706

[B19] Kang MJ, Rangel A, Camus M, Camerer CF (2011) Hypothetical and real choice differentially activate common valuation areas. J Neurosci 31:461–468. 10.1523/JNEUROSCI.1583-10.2011 21228156 PMC6623437

[B20] List JA, Gallet CA (2001) What experimental protocol influence disparities between actual and hypothetical stated values? Environ Resource Econ (Dordr) 20:241–254. 10.1023/A:1012791822804

[B21] Little J, Berrens R (2004) Explaining disparities between actual and hypothetical stated values: further investigation using meta-analysis. Econ Bull 3:1–13.

[B22] Logan GD (1994) On the ability to inhibit thought and action: a users’ guide to the stop signal paradigm In: Inhibitory processes in attention, memory, and language (Dagenbach D, Carr TH, eds), pp 189–239. San Diego, CA: Academic.

[B23] Malik VS, Pan A, Willett WC, Hu FB (2013) Sugar-sweetened beverages and weight gain in children and adults: a systematic review and meta-analysis. Am J Clin Nutr 98:1084–1102. 23966427 10.3945/ajcn.113.058362 PMC3778861

[B24] Medic N, Ziauddeen H, Vestergaard MD, Henning E, Schultz W, Farooqi IS, Fletcher PC (2014) Dopamine modulates the neural representation of subjective value of food in hungry subjects. J Neurosci 34:16856–16864. 10.1523/JNEUROSCI.2051-14.2014 25505337 PMC4261106

[B25] Mischel W, Moore B (1973) Effects of attention to symbolically presented rewards on self-control. J Pers Soc Psychol 28:172–179. 4747220 10.1037/h0035716

[B26] Morenga LT, Mallard S, Mann J (2013). Dietary sugars and body weight: systematic review and meta-analyses of randomised controlled trials and cohort studies. BMJ 346:e7492. 23321486 10.1136/bmj.e7492

[B27] Murphy JJ, Allen PG, Stevens TH, Weatherhead D (2005) A meta-analysis of hypothetical bias in stated preference valuation. Environ Resour Econ (Dordr) 30:313–325. 10.1007/s10640-004-3332-z

[B28] National Obesity Observatory (2012). Adult obesity and socioeconomic status. Oxford: National Obesity Observatory.

[B29] Nederkoorn C, Houben K, Hofmann W, Roefs A, Jansen A (2010) Control yourself or just eat what you like? Weight gain over a year is predicted by an interactive effect of response inhibition and implicit preference for snack foods. Health Psychol 29:389–393. 10.1037/a0019921 20658826

[B30] O’Brien G, Davies M (2007) Nutrition knowledge and body mass index. Health Educ Res 22:571–575. 17041019 10.1093/her/cyl119

[B31] Otter T, Johnson J, Rieskamp J, Allenby GM, Brazell JD, Diederich A, Hutchinson JW, MacEachern S, Ruan S, Townsend J (2008) Sequential sampling models of choice: some recent advances. Mark Lett 19:255–267. 10.1007/s11002-008-9039-0

[B32] Patton JH, Stanford MS, Barratt ES (1995) Factor structure of the Barratt impulsiveness scale. J Clin Psychol 51:768–774. 8778124 10.1002/1097-4679(199511)51:6<768::aid-jclp2270510607>3.0.co;2-1

[B45] Pinheiro J, Bates D, DebRoy S, Sarkar D, Team RC. nlme: Linear and nonlinear mixed effects models, 2012 R package version;3:103.

[B33] Pinheiro J, Bates D, Sarkar D (2013) nlme: linear and nonlinear mixed effects modelsnlme: linear and nonlinear mixed effects models.

[B34] Plassmann H, O’Doherty J, Rangel A (2007) Orbitofrontal cortex encodes willingness to pay in everyday economic transactions. J Neurosci 27:9984–9988. 10.1523/JNEUROSCI.2131-07.2007 17855612 PMC6672655

[B35] Roberts K, Marvin K (2011) Knowledge and attitudes towards healthy eating and physical activity: what the data tell us. Oxford: National Obesity Observatory.

[B36] Rollins BY, Dearing KK, Epstein LH (2010) Delay discounting moderates the effect of food reinforcement on energy intake among non-obese women. Appetite 55:420–425. 10.1016/j.appet.2010.07.014 20678532 PMC3042851

[B37] Saelens BE, Epstein LH (1996) Reinforcing value of food in obese and non-obese women. Appetite 27:41–50. 10.1006/appe.1996.0032 8879418

[B38] Schachter S, Rodin J (1974). Obese humans and rats. Potomac, MD: Erlbaum Associates.

[B39] Temple JL, Legierski CM, Giacomelli AM, Salvy S-J, Epstein LH (2008) Overweight children find food more reinforcing and consume more energy than do nonoverweight children. Am J Clin Nutr 87:1121–1127. 18469229 10.1093/ajcn/87.5.1121 PMC4185183

[B40] Tetley A, Brunstrom J, Griffiths P (2009) Individual differences in food-cue reactivity. The role of BMI and everyday portion-size selections. Appetite 52:614–620. 10.1016/j.appet.2009.02.005 19501758

[B41] UK Food Standards Agency (2009). Attitudes and behaviours towards healthy eating and food safety: a scoping study. London: Policy Studies Institute This is an online document, available at: http://www.food.gov.uk/sites/default/files/multimedia/pdfs/foodandyouscoping.pdf.

[B42] van Strien T, Frijters JER, Bergers GPA, Defares PB (1986) The Dutch Eating Behavior Questionnaire (DEBQ) for assessment of restrained, emotional, and external eating behavior. Int J Eat Disord 5:295–315. 10.1002/1098-108X(198602)5:2<295::AID-EAT2260050209>3.0.CO;2-T

[B43] Venables WN, Ripley BD (2002) Modern applied statistics with S. New York: Springer.

[B44] Woelbert E, Goebel R (2013) Temptation in economic decision making: effects of immediate reward and reward-cues. Neurosci Neuroecon 2:11–19.

